# Prognostic factors of IgA nephropathy presenting with mild proteinuria at the time of diagnosis (a multicenter cohort study)

**DOI:** 10.1007/s10157-023-02316-2

**Published:** 2023-01-27

**Authors:** Sayuri Shirai, Takashi Yasuda, Hiroo Kumagai, Hanako Matsunobu, Daisuke Ichikawa, Yugo Shibagaki, Yoshinari Yasuda, Keiichi Matsuzaki, Keita Hirano, Tetsuya Kawamura, Yusuke Suzuki, Shoichi Maruyama

**Affiliations:** 1 Division of Nephrology and Hypertension, Department of Internal Medicine, St. Marianna University School of Medicine, Kawasaki, Japan; 2 Department of Internal Medicine, Kichijoji Asahi Hospital, Tokyo, Japan; 3 Department of Nephrology and Endocrinology, National Defense Medical College, Tokorozawa, Saitama Japan; 4 Division of Nephrology, Self-Defense Forces Central Hospital, Tokyo, Japan; 5 Department of Nephrology/CKD Initiatives, Nagoya University Graduate School of Medicine, Nagoya, Japan; 6 Kyoto University Health Service, Kyoto, Japan; 7 Division of Nephrology, Department of Internal Medicine, Ashikaga Red Cross Hospital, Ashikaga, Japan; 8 Division of Nephrology and Hypertension, Department of Internal Medicine, Jikei University School of Medicine, Tokyo, Japan; 9 Division of Nephrology, Department of Internal Medicine, Faculty of Medicine, Juntendo University, Tokyo, Japan; 10 Division of Nephrology, Department of Internal Medicine, Faculty of Medicine, University of Nagoya, Nagoya, Japan

**Keywords:** IgA nephropathy, Mild proteinuria, Renal prognosis

## Abstract

**Background:**

Clinical factors affecting renal prognosis in patients with immunoglobulin A nephropathy (IgAN) and low urinary protein excretion (U-Prot) remain unclear. This study evaluated such factors in patients with clinical grade I (CG-I) IgAN with U-Prot < 0.5 g/day.

**Methods:**

This secondary analysis of a previous retrospective study included 394 patients with CG-I IgAN. The primary outcome was the first occurrence of a 1.5-fold increase in serum creatinine levels from baseline. Factors related to renal prognosis were examined using univariate and multivariate Cox regression analyses. CG-I was divided into C-Grade Ia (CG-Ia) (*n* = 330) with baseline eGFR ≥ 60 ml/min/1.73 m^2^, and C-Grade Ib (CG-Ib) (*n* = 64) with baseline eGFR < 60 ml/min/1.73 m^2^. Outcome incidence was compared between conservative and aggressive therapy (corticosteroids and/or tonsillectomy) groups.

**Results:**

Overall outcome incidence was significantly higher in CG-Ib than in CG-Ia; the cumulative incidence was significantly higher in CG-Ib (hazard ratio, 9.67; 95% confidence interval, 2.90–32.23). Older age, higher IgA levels, eGFR < 60 mL/min/1.73 m^2^, lower eGFR at baseline were independent prognostic factors for CG-I. Older age, lower eGFR, higher IgA levels at baseline, and U-Prot remission at 1-year post-diagnosis were independent prognostic factors for CG-Ib. Aggressive therapy tended to suppress the cumulative outcome incidence compared with conservative therapy in CG-Ib (*p* = 0.087).

**Conclusion:**

An eGFR < 60 mL/min/1.73 m^2^ is a significant predictor of renal prognosis in patients with IgAN and U-Prot < 0.5 g/day.

**Supplementary Information:**

The online version contains supplementary material available at 10.1007/s10157-023-02316-2.

## Introduction

IgA nephropathy (IgAN) is the most common primary glomerular disease and is an important cause of renal failure. Approximately, 30–40% of patients progress to end-stage renal disease (ESRD) within 20 years. Many previous studies have reported associations between the long-term prognosis in patients with IgAN and both clinical and histological parameters [1–7].

The Oxford classification is widely used for the evidence-based determination of IgAN histological severity. Nevertheless, the clinical and histological grading criteria developed by the Japanese Society of Nephrology (described in the third version of the Clinical Practice Guides for IgAN) are more frequently used in Japan [6, 7]. Based on these criteria, the clinical grade (CG) of IgAN can be classified into three categories according to the amount of daily urinary protein excretion (U-Prot) and estimated glomerular filtration rate (eGFR) at the time of renal biopsy: CG-I (U-Prot < 0.5 g/day); CG-II (U-Prot ≥ 0.5 g/day and eGFR ≥ 60 mL/min/1.73 m^2^); and CG-III (U-Prot ≥ 0.5 g/day and eGFR < 60 mL/min/1.73 m^2^) [6, 7]. While CG-I is considered to be a mild form of the disease, it can lead to end-stage renal failure in some cases [8–11]. However, there are few reports on the long-term renal prognosis in IgAN patients with mild proteinuria. The present study focused on the long-term renal prognosis of those patients.

This study was a secondary analysis of a previous nationwide multicenter study conducted in Japan (the Japanese Nationwide Retrospective Cohort Study in IgAN [JNR-IgAN]) [12]. Our objective was to evaluate the potential associations of long-term renal prognosis with the clinical background, initial therapies, and post-treatment urinary findings in patients with IgAN classified as CG-I.

## Materials and methods

### Participant population and data collection

**Fig. 1:**
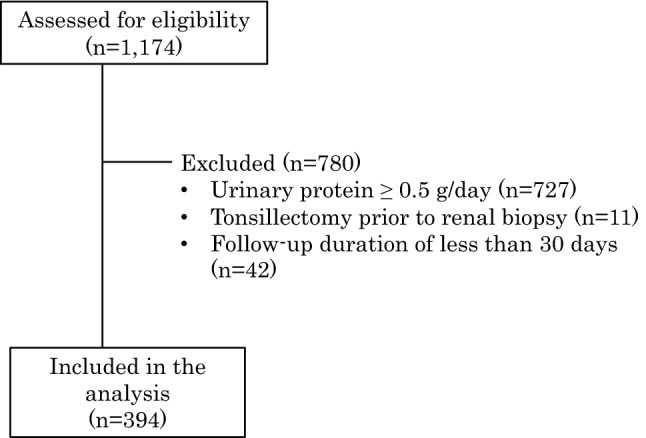
Flow chart of the participant selection process

This study was a part of a project conducted by the Progressive Kidney Disease Study Group, which was funded by the Japanese Ministry of Health, Labour and Welfare. The study protocol was approved in 2012 by the St. Marianna University School of Medicine Institutional Review Board on Human Research (which served as the main committee for ethics approval), as well as the local ethics committee of each participating institute. The data for this nationwide retrospective study were obtained from the JNR-IgAN database. All patients with IgAN were eligible for inclusion in the JNR-IgAN cohort if they were older than 18 years and had received a diagnosis of IgAN via an initial renal biopsy conducted between 2002 and 2004. A total of 1174 patients with IgAN from 42 universities and leading community hospitals within major cities across Japan were registered in the JNR-IgAN cohort. Among these patients, 394 had a baseline urinary protein excretion (U-Prot) < 0.5 g/day; we excluded patients who underwent a tonsillectomy prior to renal biopsy, as well as those with an observation period of ≤ 30 days or missing data (baseline serum creatinine level, treatment received after diagnosis, and outcomes) (Fig. 1). The time frame for registration was August 2012 to April 2013, and various clinical data were collected at the time of renal biopsy and every 3 months thereafter. The final follow-up date was January 31, 2014. Analyses were conducted from May 12, 2019, to February 28, 2020, and involved the use of an anonymized dataset, as per the protocol.

### Initial treatments

Treatments administered up to 1 year after a renal biopsy were defined as initial treatments, classified as either aggressive therapy (corticosteroid therapy and/or tonsillectomy) or conservative therapy (neither corticosteroid therapy nor tonsillectomy). Aggressive therapy was further categorized as follows: (1) tonsillectomy, steroid pulses, and oral steroids; (2) tonsillectomy and oral steroids; (3) tonsillectomy alone; (4) steroid pulses and oral steroids; and (5) oral steroids alone. Conservative therapy was categorized based on whether a renin angiotensin system inhibitor (RAS-I) was used (RAS-I +) or not used (RAS-I-). RAS-I therapy was also recorded as an initial treatment if it had already been administered at the time of renal biopsy.

### Outcome

The outcome was defined as the first occurrence of a 1.5-fold increase in serum creatinine levels from baseline or dialysis induction.

### Clinical grading criteria

Clinical grading at the time of renal biopsy was performed in accordance with the criteria of the Japanese Society of Nephrology. CG-I (U-Prot < 0.5 g/day) was divided into two subgrades according to the eGFR: CG-Ia (eGFR ≥ 60 mL/min) and CG-Ib (eGFR < 60 mL/min). We calculated the survival time from renal biopsy (baseline) until the occurrence of a 1.5-fold increase in serum creatinine levels from baseline or dialysis induction (or the end of follow-up through August 1, 2013) for each clinical grading criterion.

### Statistical analysis

Baseline characteristics are summarized as means with standard deviations (SDs) or medians with interquartile ranges (IQRs) for continuous variables; categorical variables are presented as percentages. We used the Mann–Whitney and chi-squared tests for variation analyses. A Kaplan–Meier analysis, log-rank test, and Cox regression model were used to explore the association between each treatment group (or CG subgroup) and the primary outcome.

Univariate and multivariate Cox regression analyses were performed to determine whether the primary outcome was associated with baseline characteristics, initial treatments, and remission of proteinuria (U-Prot < 0.3 g/day) or hematuria (urine occult – or ±) at 1 year after initial treatment. In the multivariate analysis of renal survival in C-Grade I, age, eGFR, and eGFR of < 60 ml/min/1.73 m^2^ were analyzed separately in models A, B, and C because of their influence on one another. In the multivariate analysis of renal survival in CG-Ib, age and eGFR were analyzed separately in models A, B because of their influence on one another. All statistical tests were two sided, and the level of statistical significance was set at p < 0.05. All statistical analyses were performed using SPSS software (version 26.0, IBM Corp., Armonk, NY, USA).

## Results 

### Baseline characteristics

The baseline characteristics of the 394 patients (48.5% male; mean (SD) age, 36.5 (15.4) years) are summarized in Table 1. The mean (SD) eGFR was 86.9 (27.4) mL/min/1.73 m^2^ and the median (IQR) U-Prot was 0.20 (0.11–0.31) g/day. CG-Ia and CG-Ib were diagnosed in 330 and 64 patients, respectively. Age, body mass index (BMI), blood pressure, uric acid, and U-Prot were significantly higher in patients with CG-Ib than in those with CG-Ia.

**Table 1:** Baseline characteristics, initial therapy, and follow-up data

Variables	All (*n* = 394)	CG-Ia (*n* = 330)	CG-Ib (*n* = 64)	*P* value
Baseline characteristics				
Age, years ^a^	36.5 ± 15.4	33.0 ± 13.4	54.5 ± 12.3	< 0.001
Women, *n* (%)	203 (51.5)	177 (53.6)	26 (40.6)	0.057
BMI, kg/m^2 a^	22.1 ± 3.3	21.9 ± 3.2	23.1 ± 3.1	< 0.001
SBP, mmHg ^a^	121.3 ± 17.1	119.4 ± 16.3	131.0 ± 17.9	< 0.001
DBP, mmHg ^a^	72.9 ± 11.8	71.9 ± 11.5	78.0 ± 12.4	< 0.001
alb, g/dl ^a^	4.28 ± 0.40	4.21 ± 0.39	4.00 ± 0.37	< 0.001
Cr, mg/dl ^a^	0.80 ± 0.35	0.71 ± 0.15	1.26 ± 0.62	< 0.001
eGFR, ml/min/1.73 m^2 a^	86.9 ± 27.4	94.6 ± 22.4	47.2 ± 10.2	< 0.001
Uric acid, mg/dl ^a^	5.4 ± 1.5	5.2 ± 1.4	6.6 ± 1.6	< 0.001
IgA, mg/dl ^a^	332.2 ± 125.2	328.7 ± 120.9	351.3 ± 146.4	0.489
U-Prot, g/day ^b^	0.20 (0.11, 0.31)	0.20 (0.10, 0.30)	0.26 (0.16, 0.36)	0.008
U-OB≧2 + , *n* (%)	283 (71.8)	241 (73.0)	42 (65.6)	0.228
Initial therapy				
Aggressive therapy, *n* (%)	155 (39.3)	138 (41.8)	17 (26.6)	0.022
Tx + SP + OS	43 (10.9)	37 (11.2)	6 (9.4)	
Tx + OS	6 (1.5)	6 (1.8)	0 (0)	
Tx	33 (8.3)	31 (9.4)	2 (3.1)	
SP + OS	16 (4.1)	11 (3.3)	5 (7.8)	
OS	57 (14.5)	53 (16.1)	4 (6.3)	
Conservative therapy, *n* (%)	239 (60.7)	192 (58.2)	47 (73.4)	0.022
RAS-I, *n* (%)	174 (44.2)	124 (37.6)	50 (78.1)	< 0.001
Aggressive therapy, *n* (%)	61 (39.4)	50 (36.2)	11 (64.7)	0.025
Conservative therapy, *n* (%)	113 (47.3)	74 (38.5)	39 (83.0)	< 0.001
Follow-up				
Duration, years ^b^	5.7 (1.8, 8.6)	5.6 (1.7, 8.4)	6.1 (2.2, 8.8)	0.473
Patients who reached outcomes,* n *(%)	12 (3)	4 (1.2)	8(12.5)	< 0.001

*RAS-I* treatment with renin angiotensin system inhibitor including adding on other treatment

*CG* C-grade, *BMI* body mass index, *SBP* systolic blood pressure, *DBP* diastolic blood pressure, *alb* albumin, *Cr* creatinine, *U-Prot* urinary protein excretion, *U-OB* urinary occult blood, *eGFR* estimated glomerular filtration rate, *Tx* tonsillectomy, *SP* steroid pulse, *OS* oral steroid

Aggressive therapy; treatment with steroid and/or tonsillectomy

Conservative therapy; treatment with neither steroid nor tonsillectomy

^a^Mean ± SD

^b^Median (interquartile range, IQR)

### Initial treatments and follow-up

Table 1 provides a detailed overview of the different types of aggressive therapies administered in 155 (39.3%) patients. Patients with CG-Ia underwent aggressive therapy more frequently compared to those with CG-Ib (41.8% versus 26.6%, *p* = 0.022). Conservative therapy was administered in 239 (60.7%) patients. Among the initial therapies, RAS-I was administered in 174 (44.2%) patients. The rate of RAS-I administration was significantly higher in patients with CG-Ib than in those with CG-Ia (78.1% versus 37.6%, *p* < 0.001).

A 1.5-fold increase in serum creatinine levels from baseline or dialysis induction was observed in 12 patients (3.0%) over a median (IQR) follow-up period of 5.7 (1.8–8.6) years. The outcome was significantly more frequently documented in patients with CG-Ib than in those with CG-Ia (12.5% versus 1.2%, *p* < 0.001) (Table 1).

### Comparison of the cumulative incidence of the outcome between patients with CG-Ia and CG-Ib

**Fig. 2:**
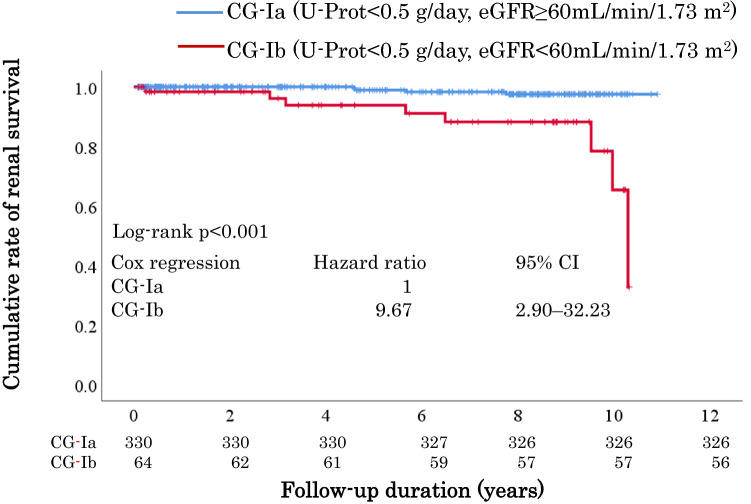
Comparison of the cumulative incidence of the first occurrence of a 1.5-fold increase in serum creatinine levels, from baseline or dialysis induction, between patients with CG-Ia and CG-Ib. *CG* clinical grade, *CI* confidence interval, *eGFR* estimated glomerular filtration rate, *U-Prot* urinary protein excretion

The Kaplan–Meier survival curve indicated that renal survival in patients with CG-Ib was significantly worse than that in patients with CG-Ia (hazard ratio [HR], 9.67; 95% confidence interval [CI], 2.90–32.23; *p* < 0.001) (Fig. 2). The renal survival rates for CG-Ia and CG-Ib were 98.8% and 87.5%, respectively.

### Factors associated with the outcome in patients with CG-I

Univariate analyses found that the outcome was associated with eGFR < 60 mL/min/1.73 m^2^, lower albumin levels, higher uric acid and IgA levels, as well as older age at baseline. Proteinuria remission at 1-year post-diagnosis was significantly associated with a reduced risk of the outcome. In contrast, the outcome was not associated with the degree of hematuria at baseline, remission of hematuria after 1 year, or the administration of aggressive therapy. Multivariate analysis showed that older age, lower eGFR, and higher IgA levels were independent predictors of the outcome (age: HR, 1.10; 95% CI 0.99–1.21; *p* < 0.1 in multivariate Model A, eGFR: HR, 0.91; 95% CI 0.83–0.99; *p* < 0.05 in multivariate Model B, IgA: HR, 1.01; 95% CI 1.00–1.02; *p* < 0.05 in multivariate Model A, Model B and Model C). An eGFR < 60 mL/min/1.73 m^2^ was a particularly strong risk factor in multivariate Model C (HR, 19.27; 95% CI 1.21–305.79; *p* < 0.05) (Table 2).

**Table 2:** Univariate and multivariate analyses for renal survival in C-Grade I (CG-I)

Variables	Univariate HR (95% CI)	Multivariate HR (95% CI)		
		Model A	Model B	Model C
Baseline characteristics				
Age, years	1.06 (1.02–1.10)***	1.10 (0.99–1.21)*		
Male vs. female	3.57 (0.96–13.22)			
BMI, kg/m^2^	1.02 (0.86–1.21)			
SBP, mmHg	1.02 (0.99–1.05)			
DBP, mmHg	0.98 (0.93–1.03)			
alb, g/dl	0.29 (0.12–0.67)***	2.08 (0.17–25.69)	2.96 (0.23–38.84)	1.91(0.16–22.42)
eGFR, ml/min/1.73m^2^	0.98 (0.96- 1.00)*		0.91 (0.83–0.99)**	
eGFR < 60 ml/min/1.73m^2 ※^	9.67 (2.90–32.2)***			19.27(1.21–305.79)**
Uric acid, mg/dl	1.55 (1.06–2.25)**	1.87 (0.77–4.55)	1.36 (0.49–3.77)	1.74(0.67–4.54)
IgA, mg/dl	1.00 (1.00–1.01)**	1.01 (1.00–1.02)*	1.01 (1.00–1.02)**	1.01(1.00–1.02)**
Log U-Prot	5.55 (0.62–49.9)			
U-OB≧2 + ^※^	1.41 (0.18–10.94)			
Initial treatment				
RAS-I ^※^	2.33 (0.70–7.74)			
Aggressive therapy^※^	0.35 (0.08–1.58)			
Clinical course				
1 year after the diagnosis				
U-Prot < 0.3 g/day^※^	0.05 (0.01–0.36)**	♰	♰	♰
U-OB − or ±	0.27 (0.03–2.21)			

Aggressive therapy; treatment with steroid and/or tonsillectomy

RAS-I; treatment with renin angiotensin system inhibitor including adding on other treatment

*HR* hazard ratio, *BMI* body mass index, *SBP* systolic blood pressure, *DBP* diastolic blood pressure, *alb* albumin, *U-Prot* urinary protein excretion, *U-OB* urinary occult blood, *eGFR* estimated glomerular filtration rate

*P* < 0.1*, P < 0.05 **, P < 0.01***, ^※^ *Yes versus No*

♰Confidence intervals (CIs) could not be calculated because the number of patients included as the reference group was extremely small

### Factors associated with the outcome in patients with CG-Ib

Univariate analyses showed that a lower albumin, a lower eGFR, and higher IgA levels at baseline were significantly associated with the outcome. The remission of proteinuria at 1 year after diagnosis was significantly associated with a reduced risk of the outcome. Multivariate Cox regression analysis showed that older age, lower eGFR, higher IgA levels at baseline, and remission of proteinuria at 1 year after diagnosis were independent predictors of the outcome (eGFR: HR, 0.90; 95% CI 0.86–0.95; *p* < 0.01, IgA: HR, 1.01; 95% CI 1.00–1.02; *p* < 0.01, and the remission of proteinuria at 1 year after diagnosis HR, 0.04; 95% CI 0.01–0.29; *p* < 0.01 in multivariate Model A, age: HR, 1.12; 95% CI 1.05–1.20; *p* < 0.01, IgA: HR, 1.01; 95% CI 1.00–1.01; *p* < 0.1, and the remission of proteinuria at 1 year after diagnosis HR, 0.04; 95% CI 0.00–0.33; *p* < 0.01 in multivariate Model B) (Table 3). The primary outcome was not significantly associated with the degree of hematuria, U-Prot at baseline, remission of hematuria at 1-year post-diagnosis, or administration of aggressive therapy.

**Table 3:** Univariate and multivariate analyses for renal survival in C-Grade Ib (CG-Ib)

Variables	Univariate HR (95% CI)	Multivariate HR (95%CI)	
		Model A	Model B
Baseline characteristics			
Age, years	1.04 (0.97–1.11)		1.12 (1.05–1.20)***
Male vs. female	2.09 (0.41–10.49)		
BMI, kg/m^2^	0.99 (0.77–1.28)		
SBP, mmHg	1.01 (0.97–1.05)		
DBP, mmHg	0.97 (0.91–1.02)		
alb, g/dl	0.20 (0.04- 0.88)**	3.49 (0.62–19.58)	2.76 (0.45–17.04)
eGFR, ml/min/1.73 m^2^	0.93 (0.87–1.00)**	0.90 (0.86–0.95)***	
Uric acid, mg/dl	1.22 (0.72–2.09)		
IgA, mg/dl	1.01 (1.00–1.01)***	1.01 (1.00–1.02)***	1.01 (1.00–1.01)*
Log U-Prot	1.48 (0.07–32.22)		
U-OB ≧ 2 + ^※^	1.23 (0.25–6.10)		
Initial treatment			
RAS-I ^※^	1.75 (0.22–14.22)		
Aggressive therapy^※^	0.03 (0.00–23.78)		
Clinical course			
1 year after the diagnosis			
U-Prot < 0.3 g/day^※^	0.11 (0.02–0.52)***	0.04 (0.01–0.29)***	0.04 (0.00- 0.33)***
U-OB–or ± ^※^	0.19 (0.02–1.65)		

RAS-I; treatment with renin–angiotensin system inhibitor including adding on other treatment

Aggressive therapy; treatment with steroid and/or tonsillectomy

*HR* hazard ratio, *CI* confidence interval, *BMI* body mass index, *SBP* systolic blood pressure, *DBP* diastolic blood pressure, *alb* albumin, *U-Prot* urinary protein excretion, *U-OB* urinary occult blood, *eGFR* estimated glomerular filtration rate

*P* < 0.1*, P < 0.05 **, P < 0.01***, ^※^*Yes versus No*

### Comparison of the cumulative incidence of the primary outcome between the aggressive therapy and conservative therapy groups in patients with CG-Ia and CG-Ib

The background comparative data of the aggressive or non-aggressive therapy group in both CG-Ia and CG-Ib are presented in the Supplementary table. There were no significant differences in the background data between the two groups, except for a higher frequency of female in the aggressive therapy group in CG-Ia.

**Fig. 3:**
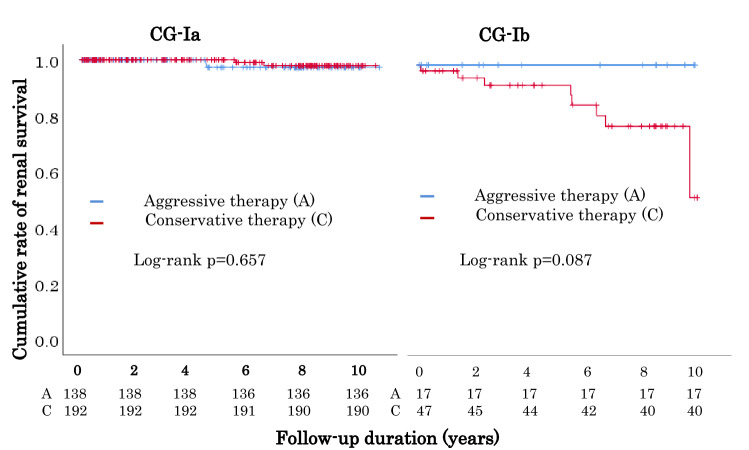
Comparison of the cumulative incidence of the first occurrence of a 1.5-fold increase in serum creatinine levels, from baseline or dialysis induction, between the aggressive therapy and conservative therapy groups among patients with CG-Ia and CG-Ib. *CG* clinical grade

Kaplan–Meier analysis did not show a significant difference in the cumulative incidence of the primary outcome between the aggressive and conservative therapy groups in patients with CG-Ia (log-rank test, *p* = 0.657). In contrast, the cumulative incidence of the primary outcome in the conservative therapy group was considerably smaller than that in the aggressive therapy group in patients with CG-Ib; however, this difference did not reach statistical significance (log-rank test, *p* = 0.087) (Fig. 3).

## Discussion

In this study, we revealed that an eGFR < 60 mL/min/1.73 m^2^ is a significant predictor of renal prognosis in patients with IgAN with U-Prot < 0.5 g/day. Prior studies have identified several factors associated with renal prognosis in patients with IgAN [1–7, 13, 14]. However, there are few reports on the long-term renal prognosis in patients with mild IgAN and mild proteinuria [8–11]. Gutierrez et al. conducted an observational study of 141 patients with IgAN who had an eGFR of > 60 mL/min/1.73 m^2^ and a proteinuria of < 0.5 g/day on initial presentation. Over a median follow-up of 108 months, serum creatinine levels increased by > 50% and > 100% in five (3.5%) patients and one (0.7%) patient, respectively; no patient developed ESRD [8]. Szeto et al. conducted a 7-year observational study of 72 patients with IgAN who presented with hematuria and minimal proteinuria (< 0.4 g/day) at the time of renal biopsy. They found that only five (7%) patients developed impaired renal function [9]. We have previously reported that in a study of 62 patients with IgAN treated conservatively, spontaneous remission of hematuria and proteinuria was achieved in about 30% of cases, and in those with urinary protein < 1 g/day, eGFR did not decrease by more than 30% within 3 years [15]. In contrast, Lee et al. documented four mortalities and six cases of ESRD in a 95-month observational study of 153 patients with IgAN, who presented with an eGFR of ≥ 60 mL/min/1.73 m^2^ and a proteinuria of < 0.5 g/day at the time of renal biopsy. The 30-year renal survival rate was 85.5%, thus suggesting that the long-term prognosis of clinically early IgAN is not always favorable [10]. Similarly, Shen et al. investigated 177 patients with early IgAN who presented with a proteinuria of ≤ 0.4 g/day and normal renal function; 43 (24%) patients developed renal insufficiency over a mean follow-up period of 111 months [11]. As conflicting results have been reported among patients with mild IgAN, there is a need for additional studies to clarify the long-term renal prognosis in patients with IgAN classified as CG-I (U-Prot < 0.5 g/day).

Since the majority of the multivariate analyses conducted in prior studies have identified eGFR as an independent predictor of renal prognosis, we compared this outcome between patients with CG-Ia and CG-Ib. Although the number of patients with CG-Ib was relatively small, we found that age, blood pressure, BMI, uric acid levels, U-Prot, and the proportion of patients administered RAS-I were significantly higher in patients with CG-Ib than in those with CG-Ia. The log-rank test indicated that patients with CG-Ib had a significantly poorer renal prognosis compared to those with CG-Ia. In addition, multivariate analysis revealed that an eGFR < 60 mL/min/1.73 m^2^ at baseline was independently associated with the primary outcome in patients with CG-I. Similarly, in CG-Ib patients, lower eGFR was independently associated with the primary outcome.

A meta-analysis of nine studies published between 1980 and 2012 found that older patients with IgAN had a 1.95 times higher risk of progression to end-stage renal failure compared to non-older patients with IgAN. Furthermore, the effect of aging on renal prognosis in patients with IgAN was greater among studies conducted in Asia than in Europe [16]. These results are consistent with our identification of aging as a significant independent predictor of poor renal prognosis in patients with CG-I and CG-Ib. Physicians are often hesitant to apply steroid therapy to elderly IgA nephropathy patients with impaired renal function whose renal pathology sometimes shows sclerotic lesions. However, the results of the present analysis, although not including histological data, suggest that steroid therapy may be effective in CG-Ib patients with possible acute glomerular lesions.

Several previous studies have reported a significant relationship between IgA/complement component 3 (C3) levels and renal prognosis in patients with IgAN. Komatsu et al. found that the serum IgA/C3 ratio appeared to reflect the histological severity of IgAN and could serve as a marker of IgAN progression in Japanese patients [17]. In a study conducted in Europe, Stefan et al. reported that patients with an IgA/C3 ratio of < 2.9 tended to exhibit a higher renal survival rate [18]. However, we were unable to evaluate baseline IgA/C3 serum levels in the present study, as many institutes often did not measure serum C3 levels at the time of diagnosis. Nevertheless, the results of these prior studies partially support our identification of the serum IgA level as an independent predictor of renal prognosis.

In the present study, U-Prot remission within 1 year after diagnosis was an independent predictor of renal prognosis in patients with CG-Ib. This finding is supported by many prior studies that have reported a favorable renal prognosis in patients with IgAN who have achieved a complete or partial remission of proteinuria [10, 19, 20]. For example, in a long-term retrospective observational study of 1154 patients with pediatric IgAN, Suh et al. found that proteinuria remission was an independent prognostic factor that was negatively correlated with progression to chronic kidney disease [21]. Thus, current evidence indicates that achieving U-Prot < 0.3 g/day may improve renal prognosis in patients with mild proteinuria at baseline.

There was no significant difference in renal prognosis between the aggressive and conservative therapy groups among patients with CG-Ia. In contrast, there was a considerable difference in renal prognosis between the two therapy groups in CG-Ib, although this did not reach statistical significance for the follow-up period in the present study. Further extension of the observation period may lead to a favorable prognosis (Fig. 3).

### Limitations

This study had several limitations. First, this was a cohort study, and patients who received additional treatment during the follow-up period were also included in the analysis. Therefore, this may have confounded the observed relationship between initial treatment and renal prognosis. Second, laboratory data regarding urinalysis parameters and renal function were only available for the last observation. Therefore, the effect of IgAN recurrence on the primary outcome remains unknown. Third, the number of patients reaching the primary endpoint was small because of the relatively low level of proteinuria at baseline; furthermore, the treatment options for each case differed depending on the attending physician. These may have masked any difference in renal prognosis between the aggressive and conservative therapy groups. Fourth, we were unable to incorporate renal biopsy findings as covariates in the Cox regression analysis because of the lack of histological data in many patients. Therefore, we could not rule out the possibility that differences in histological severity may have affected renal prognosis.

## Conclusions

The results of this nationwide Japanese retrospective cohort study indicate that an eGFR < 60 mL/min/1.73 m^2^ is an independent predictor of renal prognosis in patients with IgAN classified as CG-I (U-Prot < 0.5 g/day). Thus, the poor prognosis in patients with eGFR < 60 ml/min/1.73 m^2^ despite urinary protein excretion of < 0.5 g/day should be taken into account when it is considered necessary to revise the current classification of CG.

## Supplementary Information

Below is the link to the electronic supplementary material.

Supplementary file1 (DOCX 26 KB)
