# Supplementary material for: Prognostic factors of IgA nephropathy presenting with mild proteinuria at the time of diagnosis (a multicenter cohort study)
Source: Clin Exp Nephrol. 2023 Jan 27;27(4):340–8. doi: 10.1007/s10157-023-02316-2 (PMC10023643; doi:10.1007/s10157-023-02316-2)
Supplement: Supplementary file 1 — Supplementary file1 (DOCX 26 KB) [file 10157_2023_2316_MOESM1_ESM.docx]

Supplementary table.

| **CG-Ia (n = 330)** | **Aggressive therapy (n = 138)** | **Conservative therapy (n = 192)** |  |
| --- | --- | --- | --- |
| **Variables** |  |  | **P Value** |
| **Baseline characteristics** |  |  |  |
| Age, years ^a^ | 31.7± 13.4 | 34.0± 13.4 | 0.93 |
| Women, n(%) | 84 (60.9) | 93 (48.4) | 0.03 |
| BMI, kg/m^2^ ^a^ | 21.6± 3.1 | 22.2± 3.3 | 0.48 |
| SBP, mmHg ^a^ | 118.1± 16.5 | 120.7 ± 16.2 | 0.84 |
| DBP, mmHg ^a^ | 73.1± 11.4 | 70.2 ± 11.5 | 0.58 |
| alb, g/dl a | 4.20 ± 0.40 | 4.22± 0.41 | 0.38 |
| Cr, mg/dl ^a^ | 0.70 ± 0.16 | 0.73 ± 0.15 | 0.33 |
| eGFR, ml/min/1.73 m^2^ ^a^ | 97.4 ± 26.2 | 92.2± 18.1 | 0.57 |
| Uric acid, mg/dl ^a^ | 5.1 ± 1.5 | 5.4± 1.3 | 0.83 |
| IgA, mg/dl ^a^ | 326.7 ± 122.1 | 323.3 ± 113.6 | 0.44 |
| U-Prot, g/day ^b^ | 0.22 (0.16, 0.35) | 0.20 (0.08, 0.29) | 0.23 |
| U-OB≧2+, n (%) | 105 (76.1) | 136(70.8) | 0.18 |
| **Follow-up** |  |  |  |
| **1 year after the diagnosis** |  |  |  |
|  | n = 86 | n = 138 |  |
| U-Prot < 0.3g/day, n (%) | 74 (86.0) | 103 (74.6) | 0.04 |
|  | n = 83 | n = 121 |  |
| U-OB – or ±, n (%) | 56 (67.5) | 67 (55.4) | 0.08 |
| **CG-Ib (n = 64)** | **Aggressive Therapy (n = 17)** | **Conservative Therapy (n = 47)** |  |
| **Variables** |  |  | **P Value** |
| **Baseline Characteristics** |  |  |  |
| Age, years ^a^ | 47.3 ± 13.7 | 55.6 ± 10.2 | 0.29 |
| Women, n (%) | 9 (52.9) | 17(36.2) | 0.26 |
| BMI, kg/m2 ^a^ | 22.6 ± 3.3 | 23.9 ± 3.5 | 0.41 |
| SBP, mmHg ^a^ | 122.8 ± 16.3 | 133.6 ± 18.3 | 0.56 |
| DBP, mmHg ^a^ | 74.9 ± 9.3 | 78.3 ± 13.3 | 0.17 |
| alb, g/dl ^a^ | 4.28 ± 0.40 | 3.93 ± 0.36 | 0.09 |
| Cr, mg/dl ^a^ | 1.26 ± 0.30 | 1.13 ± 0.19 | 0.21 |
| eGFR, ml/min/1.73 m^2^ ^a^ | 45.5 ± 11.3 | 50.2 ± 6.9 | 0.44 |
| Uric acid, mg/dl ^a^ | 6.1 ± 1.4 | 6.8 ± 1.7 | 0.30 |
| IgA, mg/dl ^a^ | 335.3 ± 142.0 | 360.8 ± 151.5 | 0.40 |
| U-Prot, g/day ^b^ | 0.26 (0.16, 0.40) | 0.26 (0.16, 0.34) | 0.44 |
| U-OB≧2+, n(%) | 13 (76.5) | 29 (61.7) | 0.38 |
| **Follow -up** |  |  |  |
| **1 year after the diagnosis** |  |  |  |
|  | n = 12 | n = 39 |  |
| U-Prot < 0.3g/day, n (%) | 9 (75.0) | 27 (69.2) | 0.50 |
|  | n = 12 | n = 33 |  |
| U-OB – or ±, n (%) | 9 (75.0) | 22 (66.7) | 0.44 |

BMI; body mass index, SBP; systolic blood pressure, DBP; diastolic blood pressure, alb; albumin, U-Prot; urinary protein excretion, U-OB; urinary occult blood, eGFR; estimated glomerular filtration rate

a mean ± SD, b median (interquartile range, IQR)
